# Development and validation of a predictive model for lymph node metastases in peripheral non-small cell lung cancer with a tumor diameter ≤ 2.0 cm and a consolidation-to-tumor ratio > 0.5

**DOI:** 10.3389/fonc.2025.1436771

**Published:** 2025-01-22

**Authors:** Dongyu Li, Shaolei Li, Hongbing Zhang, Chunqiu Xia, Xiaoyong Nan, Hongyu Liu, Jun Chen

**Affiliations:** ^1^ Department of Thoracic Surgery, Yuncheng Central Hospital affiliated to Shanxi Medical University, Yuncheng, China; ^2^ Department of Lung Cancer Surgery, Tianjin Medical University General Hospital, Tianjin, China; ^3^ Department of Thoracic Surgery II, Key Laboratory of Carcinogenesis and Translational Research (Ministry of Education), Peking University Cancer Hospital and Institute, Beijing, China; ^4^ Tianjin Key Laboratory of Lung Cancer Metastasis and Tumor Microenvironment, Tianjin Lung Cancer Institute, Tianjin Medical University General Hospital, Tianjin, China

**Keywords:** lung cancer, non-small-cell lung cancer, lymph node metastases, preoperative workup, prediction model

## Abstract

**Background:**

Precisely predicting lymph node metastasis (LNM) status is critical for the treatment of early non-small5-cell lung cancer (NSCLC). In this study, we developed a LNM prediction tool for peripheral NSCLC with a tumor diameter ≤ 2.0 cm and consolidation-to-tumor ratio (CTR) > 0.5 to identify patients where segmentectomy could be applied.

**Methods:**

Clinical characteristics were retrospectively collected from 435 patients with NSCLC. Logistic regression analysis of the clinical characteristics of this development cohort was used to estimate independent LNM predictors. A prediction model was then developed and externally validated using a validation cohort at another institution.

**Results:**

Four independent predictors (tumor size, CTR, pleural indentation, and carcinoembryonic antigen (CEA) values) were identified and entered into the model. The model showed good calibration (Hosmer–Lemeshow (HL) P value = 0.680) with an area under the receiver operating characteristic curve (AUC) = 0.890 (95% confidence interval (CI): 0.808–0.972) in the validation cohort.

**Conclusions:**

We developed and validated a novel and effective model that predicted the probability of LNM for individual patients with peripheral NSCLC who had a tumor diameter ≤ 2.0 cm and CTR > 0.5. This model could help clinicians make individualized clinical decisions.

## 1 Introduction

Lung cancer is the most common cancer worldwide and the leading cause of cancer-related death (1). Surgery is the treatment of choice for early non-small-cell lung cancer (NSCLC) (2, 3). The evaluation of lymph node metastasis (LNM) status prior to surgery is important because it determines subsequent treatment strategies (4, 5).

Lobectomy has been considered the standard surgical treatment for early NSCLC since its landmark trial (3). One condition for this surgical modality is that patients do not have mediastinal LNM (2). However, with ongoing research progress, the extent of surgical resection has become more refined, while LNM status requirements have become more stringent. In 2022, the Japan Clinical Oncology Group (JCOG) 0802 study reported that segmentectomy could be considered as a standard surgical procedure for peripheral NSCLC patients with a tumor diameter ≤ 2.0 cm and a consolidation-to-tumor ratio (CTR) > 0.5 (6). One of the conditions for this approach is that patients do not have LNM.

However, current examination methods in clinical practice for preoperative LNM status still have shortcomings, such as poor diagnostic performance (7–14), anatomical location-dependent issues, high associated risks, and expense (8, 15–17). Moreover, few predictive models have been developed for the specific patient population in the JCOG0802 study.

Therefore, we developed and validated a clinically useful predictive model to assess LNM probability in peripheral NSCLC patients with a tumor diameter ≤ 2.0 cm and CTR > 0.5, which could help clinicians develop clinical decisions and formulate individualized surgical protocols.

## 2 Materials and methods

We recruited a retrospective cohort of consecutive 435 patients with NSCLC who underwent surgical treatment between January 1, 2018 and October 31, 2022. The study was approved by the Institutional Review Board of our hospital.

### 2.1 Study population

**Figure 1:**
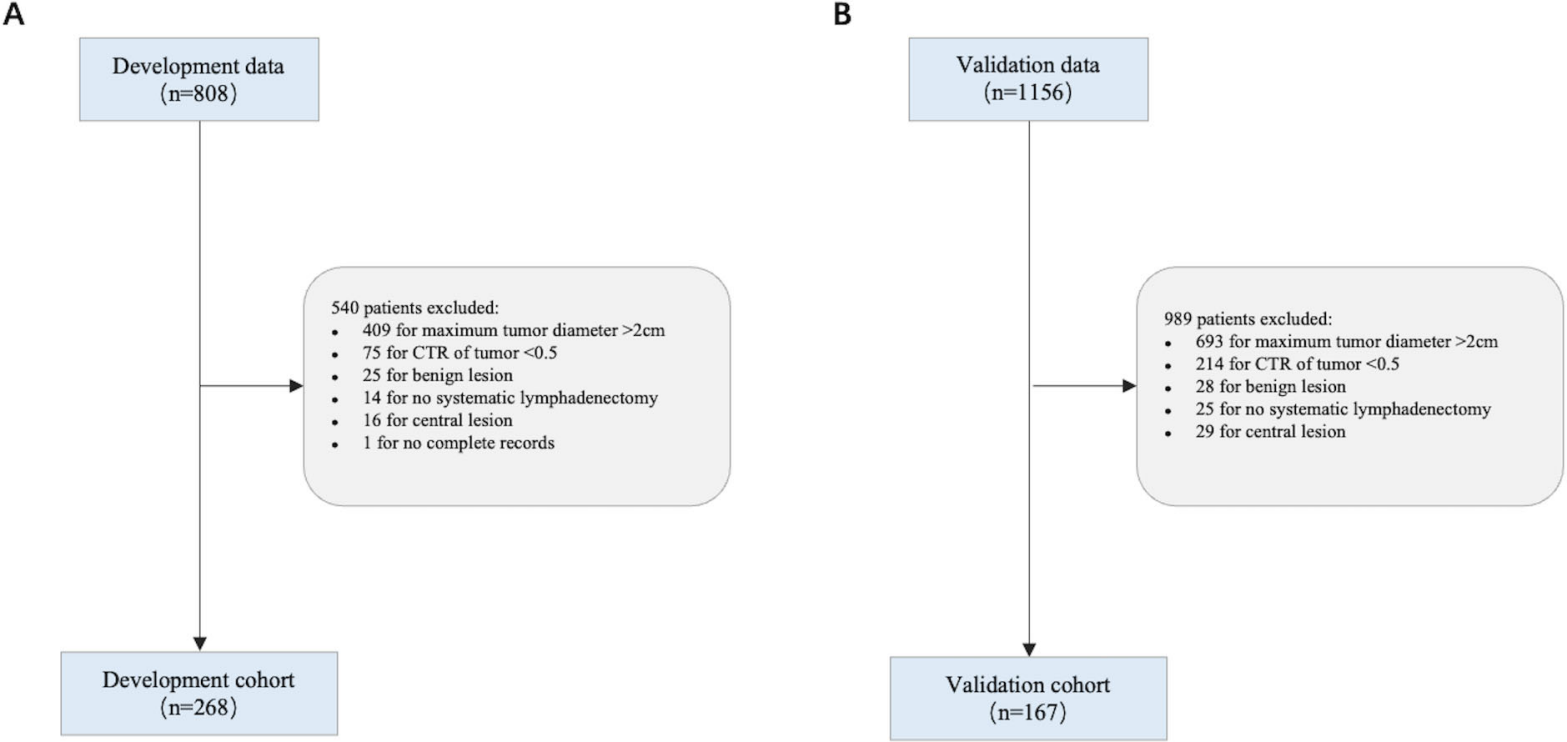
Flowchart showing the study population. **(A)** Development cohort; **(B)** Validation cohort.

The patient recruitment process is outlined (**Figure 1**). We recruited 268 cases from Yuncheng Central Hospital as the development cohort, and 167 cases from Peking University Cancer Hospital as the validation cohort.

#### 2.1.1 Inclusion criteria included

(I) patients who underwent a lobectomy or segmentectomy with systemic lymph node dissection or sampling; (II) a postoperative pathological diagnosis of primary NSCLC; (III) patients who underwent an enhanced thin layer thoracic computed tomography (CT) scan within 60 days of the operation; (IV) CT fulfilled the following criteria: single tumor, tumor center located in the outer one-third of the lung parenchyma, tumor diameter ≤ 2.0 cm, and CTR > 0.5; and (V) an age range of 20–85 years old.

#### 2.1.2 Exclusion criteria included

(I) patients having received radiotherapy, chemotherapy or a combination of both before surgery; (II) patients with simultaneous or metachronous (within the past 5 years) double cancers; (III) patients with active bacterial or fungal infections; and (IV) patients with severe pulmonary fibrosis or emphysema.

### 2.2 Outcomes

The main outcome measure was LNM-positivity as determined by pathological examination - pathological lymph node involvement (N1 or N2 lymph nodes), and vice versa for LNM-negative.

### 2.3 Predictive factors

We selected the following predictive factors that potentially influenced LNM: (I) gender (male/female); (II) age (continuous variable); (III) body mass index (BMI) (continuous variable measured in kg/m^2^); (IV) smoking history (yes/no); (V) tumor size measured by CT (≤ 1.5 cm/1.5 cm); (VI) tumor location in bronchi (fourth-order/fifth-order or higher bronchi); (VII) tumor location in the lobe (right upper lobe/right middle lobe/right lower lobe/left upper lobe/left lower lobe); (VIII) CTR (continuous variable); (IX) pleural indentation (presence/absence); and (X) carcinoembryonic antigen (CEA) value (≤ 5.0 ng/ml/> 5.0 ng/ml).

### 2.4 Surgical procedure

All surgery was performed by thoracic surgeons at respective centers. For patients with poor lung function reserve or comorbidities, and were considered to have poor surgical tolerance, segmentectomy was performed. For all other patients, lobectomy was performed. The operational criteria for systematic lymph node dissection conformed to the International Association for the Study of Lung Cancer definition of adequate lymph node dissection (18).

### 2.5 Imaging evaluations

**Figure 2:**
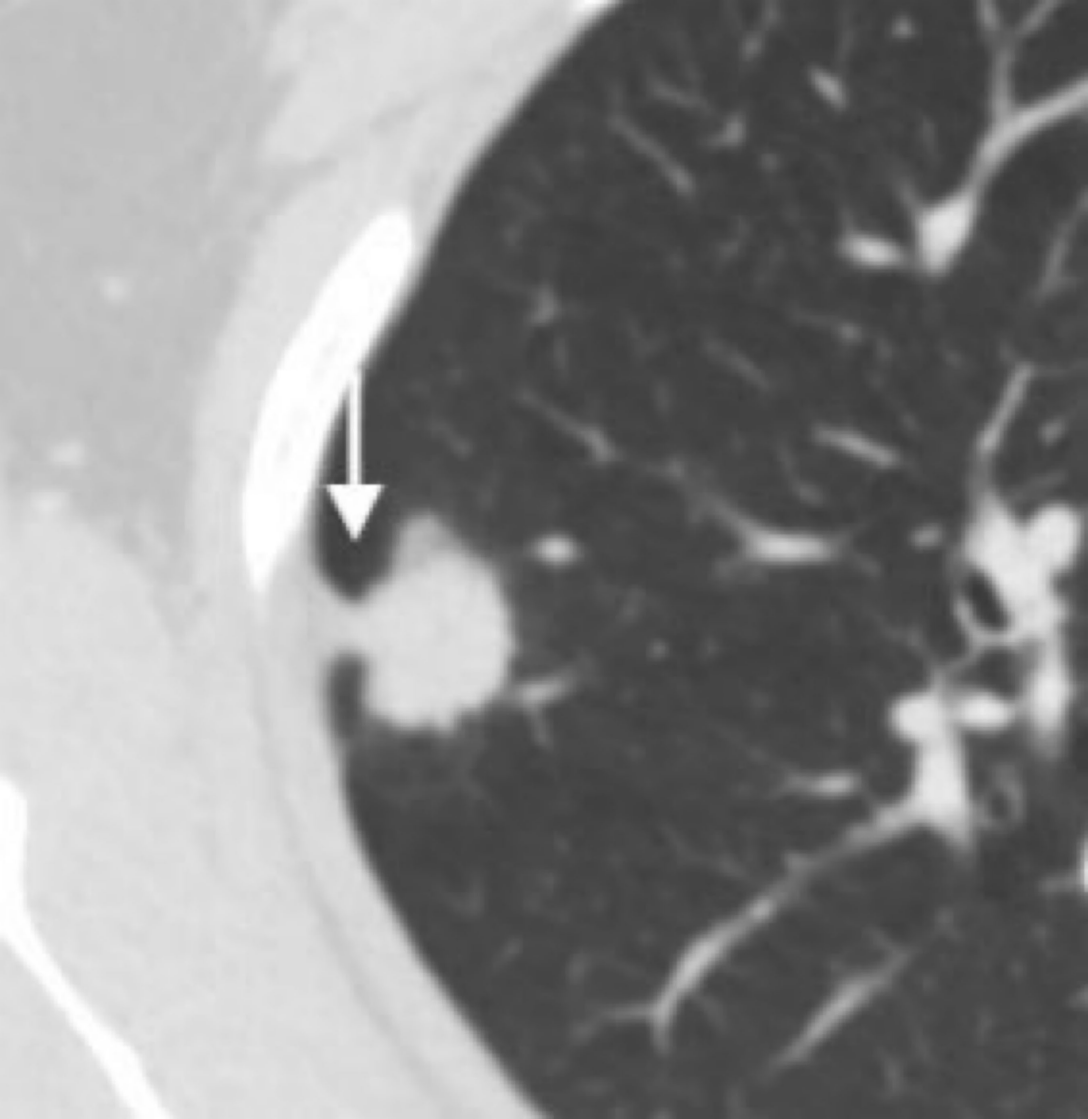
Pleural indentation (arrow).

CT images were evaluated by two independent physicians (15 and 20 years of experience, respectively) who were blinded to all patient clinical information. Tumor size, its solid component, and the presence/absence of pleural indentations, were assessed in lung window setting (window width, 1200 HU; window position, -600 HU) (19). Tumor size was defined as the largest tumor diameter, including the solid component and surrounding ground glass component on axial images. Solid component size was defined as the largest diameter of the solid tumor component on axial images. The solid component was defined as an opaque area with locally increased density such that it completely obscured underlying vascular and bronchiolar markings. The ground glass component was defined as an area with locally increased density that did not completely obscure underlying vascular structures. CTR was calculated by dividing the solid component size by the tumor size (19). Pleural indentation was defined as a visually sharp, linear indentation or cord-like linear shadow on lung tumors by CT (20, 21) (**Figure 2**).

### 2.6 Model development and validation

A predictive model was developed using development cohort data. Logistic regression analysis was performed using candidate predictors as independent variables and LNM status as a dependent variable, and correlation strength was estimated using odds ratio (OR) with 95% confidence interval (CI) values. Variables with P values < 0.1 in univariate analysis were included as candidate predictors in multivariate regression analysis, and screened using the stepwise backward method. Finally, predictors identified after screening were used to build the model, after which it was validated using validation cohort data.

Model performance was assessed by discrimination and calibration. The area under the curve (AUC) in receiver operating characteristic (ROC) analysis was used to measure discrimination, while calibration was assessed using Hosmer–Lemeshow (HL) tests and a calibration curve. Additionally, we used Brier scores to assess overall model performance, and used clinical decision curve analysis (DCA) to quantify net model benefits to patients at different threshold probabilities, thus assessing model value in clinical settings.

### 2.7 Risk stratification strategies based on clinical guideline recommendations and model performance

The relationship between the risk the stratification strategy and a pathological diagnosis is shown (**Table 1**). If a patient was pathologically diagnosed as LNM-negative but was considered high-risk by stratification, this was considered “over-screening” (Group B, **Table 1**). On the other hand, if a patient had a pathological LNM-positive diagnosis but was considered low-risk by stratification, this was considered “under-screening” (Group C, **Table 1**). From this, the stratification strategy had a sensitivity of A/(A+C); a false positive rate of B/(B+D); a specificity of D/(B+D); and a false negative rate of C/(A+C). Missed diagnoses in LNM-positive patients can have serious consequences such as tumor recurrence, so priority was given to avoid under-screening. Under this premise, a balance was sought where false positive rates were as low as possible.

**Table 1:** The relationship between the risk stratification strategy and a pathological diagnosis.

	Pathological diagnosis	
Risk stratification strategy	LNM (+)	LNM (-)
High-risk	A	B
Low-risk	C	D

LNM, lymph node metastasis.

According to American College of Chest Physicians (8) and National Comprehensive Cancer Network guidelines (2), invasive mediastinal staging is generally indicated in the following circumstances: tumor size > 3.0 cm, centrally located tumor, and the presence of lymph node disease (hilar/mediastinal lymph node enlargement, or elevated deoxyglucose uptake in lymph nodes) by imaging tests. Patients with any of these conditions are considered high-risk using these guidelines.

After modeling, we assessed the diagnostic performance of the model by setting a LNM-positive cutoff probability. We categorized patients as high-risk when their LNM-positive probability was above the cutoff probability, and vice versa for low-risk. Based on the criteria for setting the cutoff probability with different emphasis, we designed three risk stratification strategies. Strategy 1: Cutoff probability that facilitated model sensitivity to approximately that of the guideline strategy; Strategy 2: The optimal cutoff probability corresponded to the maximum Youden index in ROC analysis; and Strategy 3: Cutoff probability that maximized sensitivity (> 90%).

### 2.8 Statistical analysis

Interobserver agreement was assessed for continuous variables, categorical variables using the intraclass correlation co-efficient (ICC), and Kappa consistency tests (22). Baseline characteristics of the study population were described by mean valued ± standard deviation (normal distribution), median values with interquartile ranges (non-normal distribution) for continuous variables, and absolute and relative frequencies for categorical variables. Differences in continuous variables were compared using Mann–Whitney or two-tailed Student’s t tests. Differences in categorical variable distributions were compared using Pearson chi-square tests. Two-sided P values < 0.05 were considered statistically significant. All statistical analyses were performed in R version 4.1.1 (The R Foundation for Statistical Computing; http://www.R-project.org/).

## 3 Results

### 3.1 Interobserver agreement

Interobserver agreements were good for CT imaging features: tumor size (ICC = 0.95), solid component size (ICC = 0.94), and pleural depression (kappa = 0.92).

### 3.2 General patient characteristics

In total, 435 patients met inclusion criteria, including 184 (42.3%) males and 251 (57.7%) females, with a mean age of 59.8 years (range = 31–82 years). We observed that 59 (13.6%) patients were LNM-positive and 376 (86.4%) were LNM-negative. There were 249 (57.2%) patients with tumor sizes ≤ 1.5 cm, and 186 (42.8%) with tumor sizes > 1.5 cm. CTR was ≤ 0.75 in 153 (5.2%) patients and > 0.75 in 282 (64.8%). The demographic and clinical characteristics of both cohorts are shown (**Table 2**). The development cohort included 268 (61.6%) patients and the validation cohort included 167 (38.4%) patients. The LNM-positive rate was 14.2% (38/268) in the development cohort and 12.6% (21/167) in the validation cohort. Significant differences were observed between cohorts in terms of gender and tumor size. When compared with the development cohort, the validation cohort had a larger proportion of females (65.9% versus 52.6%, P value = 0.009) and a larger proportion of patients with tumor sizes ≤ 1.5 cm (67.1% versus 51.1%, P value = 0.002), which were possibly related to the fact that regions where validation centers were located were more economically developed, and populations may have had a better awareness of medical checkups, which helped earlier disease detection.

**Table 2:** Baseline patient characteristics.

Characteristic	Total (n=435) (%)	Development cohort (n=268) (%)	Validation cohort (n=167) (%)	*P-*value
Gender				
Male	184(42.3)	127(47.4)	57 (34.1)	0.009
Female	251(57.7)	141(52.6)	110 (65.9)	
Age, years				
Mean (SD)	59.80 (9.18)	60.36 (9.00)	58.90 (9.41)	0.106
BMI				
Mean (SD)	24.40 (3.15)	24.52 (3.16)	24.20 (3.13)	0.296
Smoking				
No	305 (70.1)	184 (68.7)	121 (72.5)	0.463
Yes	130 (29.9)	84 (31.3)	46 (27.5)	
Tumor size				
≤ 1.5 cm	249 (57.2)	137 (51.1)	112 (67.1)	0.002
>1.5 cm	186 (42.8)	131 (48.9)	55 (32.9)	
Tumor location in bronchi				
Fourth-order bronchi	148 (34.0)	86 (32.1)	62 (37.1)	0.330
Fifth-order or higher bronchi	287 (66.0)	182 (67.9)	105 (62.9)	
Tumor location in lobe				
RUL	145 (33.3)	97 (36.2)	48 (28.7)	0.483
RLL	90 (20.7)	50 (18.7)	40 (24.0)	
RML	33 (7.6)	21 (7.8)	12 (7.2)	
LUL	97 (22.3)	59 (22.0)	38 (22.8)	
LLL	70 (16.1)	41 (15.3)	29 (17.4)	
CTR				
≤0.75	153 (35.2)	92 (34.3)	61 (36.5)	0.716
>0.75	282 (64.8)	176 (65.7)	106 (63.5)	
Pleural indentation				
Absence	243 (55.9)	148 (55.2)	95 (56.9)	0.810
Presence	192 (44.1)	120 (44.8)	72 (43.1)	
CEA				
≤ 5.0 ng/ml	388 (89.2)	240 (89.6)	148 (88.6)	0.885
>5.0 ng/ml	47 (10.8)	28 (10.4)	19 (11.4)	
Surgical procedure				
Lobectomy	342 (78.6)	213 (79.5)	129 (77.2)	0.666
Segmentectomy	93 (21.4)	55 (20.5)	38 (22.8)	
Number of lymph nodes removed				
Median (IQR)	11.00 [9.00, 14.00]	12.00 [10.00, 14.00]	11.00 [8.00, 14.00]	0.335
Histological type				
Squamous cell carcinoma	12 (2.8)	10 (3.7)	2 (1.2)	0.286
Others	7 (1.6)	4 (1.5)	3 (1.8)	
Adenocarcinoma	416 (95.6)	254 (94.8)	162 (97.0)	
pN staging				
N0	376 (86.4)	230 (85.8)	146 (87.4)	0.889
N1	23 (5.3)	15 (5.6)	8 (4.8)	
N2	36 (8.3)	23 (8.6)	13 (7.8)	

SD, standard deviation; IQR, interquartile range; RUL, right upper lobe; RML, right middle lobe; RLL, right lower lobe; LUL, left upper lobe; LLL, left lower lobe.

### 3.3 Model development

Univariate analyses showed that tumor size, CTR, pleural indentation, and CEA values were significantly associated with LNM-positive tumors (P value < 0.05, **Table 3**). We then included these variables in multivariate analysis. Additionally, although the tumor location in the bronchi predictor was not statistically significant in univariate analysis, we included this in multivariate analysis due to its clinical significance (9, 23).

**Table 3:** Univariate analysis of lymph node metastasis (LNM) risk.

Characteristic	Total (n=268) (%)	LNM (-) (n=230) (%)	LNM (+) (n=38) (%)	*P-*value
Gender				
Male	127 (47.4)	106 (46.1)	21 (55.3)	0.382
Female	141 (52.6)	124 (53.9)	17 (44.7)	
Age, years				
Mean (SD)	60.36 (9.00)	60.49 (8.93)	59.61 (9.51)	0.577
BMI				
Mean (SD)	24.52 (3.16)	24.41 (3.18)	25.20 (3.00)	0.158
Smoking				
No	184 (68.7)	162 (70.4)	22 (57.9)	0.175
Yes	84 (31.3)	68 (29.6)	16 (42.1)	
Tumor size				
≤ 1.5 cm	137 (51.1)	128 (55.7)	9 (23.7)	0.001
>1.5 cm	131 (48.9)	102 (44.3)	29 (76.3)	
Tumor location in bronchi				
Fourth-order bronchi	86 (32.1)	71 (30.9)	15 (39.5)	0.387
Fifth-order or higher bronchi	182 (67.9)	159 (69.1)	23 (60.5)	
Tumor location in lobe				
RUL	97 (36.2)	87 (37.8)	10 (26.3)	0.618
RLL	50 (18.7)	40 (17.4)	10 (26.3)	
RML	21 (7.8)	18 (7.8)	3 (7.9)	
LUL	59 (22.0)	50 (21.7)	9 (23.7)	
LLL	41 (15.3)	35 (15.2)	6 (15.8)	
CTR				
≤ 0.75	92 (34.3)	88 (38.3)	4 (10.5)	0.002
>0.75	176 (65.7)	142 (61.7)	34 (89.5)	
Pleural indentation				
Absence	148 (55.2)	140 (60.9)	8 (21.1)	< 0.001
Presence	120 (44.8)	90 (39.1)	30 (78.9)	
CEA				
≤ 5.0 ng/ml	240 (89.6)	216 (93.9)	24 (63.2)	< 0.001
>5.0 ng/ml	28 (10.4)	14 (6.1)	14 (36.8)	

LNM, lymph node metastasis; SD, standard deviation; RUL, right upper lobe; RML, right middle lobe; RLL, right lower lobe; LUL, left upper lobe; LLL, left lower lobe.

**Figure 3:**
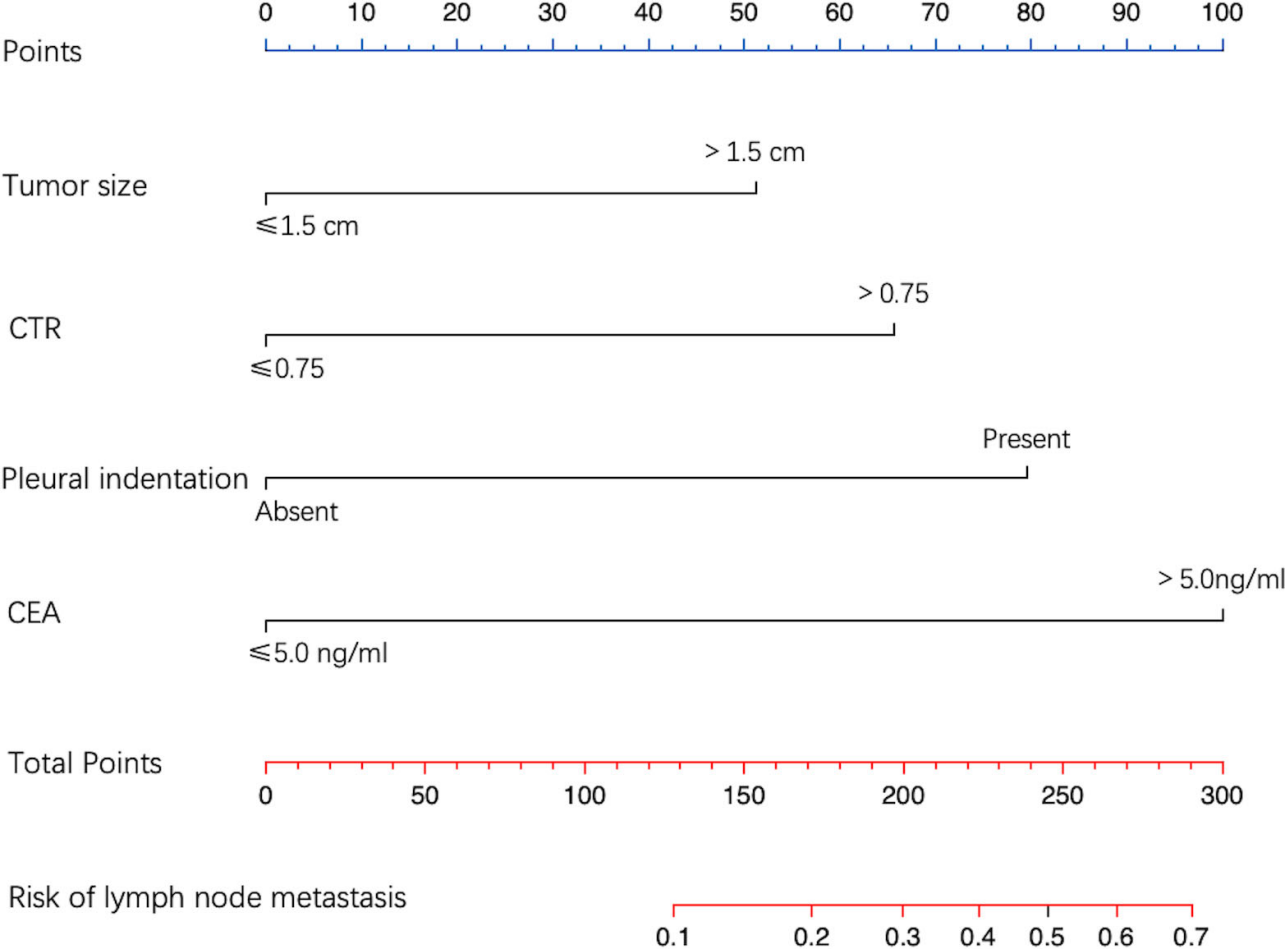
Nomogram for predicting LNM probability. The value of each predictive factor was given a score on the point scale axis. A total score was calculated by adding every single score, and by projecting the total score to the lower total point scale, LNM probability was estimated.

From multivariate logistic regression analysis, we identified four independent LNM-positive predictors: tumor size (OR = 2.613; 95% CI: 1.130–6.468; P value = 0.029), CTR (OR = 3.428; 95% CI: 1.196–12.540; P value = 0.035), pleural indentation (OR = 4.441; 95% CI: 1.899–11.499; P value = 0.001), and CEA values (OR = 6.521; 95% CI: 2.537–17.196; P value < 0.001) (**Table 4**). The model indicated that patients with a tumor size > 1.5 cm, a CTR > 0.75, a pleural indentation around the tumor by CT imaging, and a CEA value > 5.0 ng/ml had a greater risk of being LNM-positive. Based on this model, we developed a nomogram to assess the probability of a patient presenting with LNM-positive status (**Figure 3**).

**Table 4:** Variables identified by logistic multivariable regression analysis.

Variable	Multivariable analysis			Factors selected for model		
	OR	95% CI	*P-*value	OR	95% CI	*P-*value
Tumor size						
≤ 1.5 cm	Ref.			Ref.		
>1.5 cm	2.452	1.041-6.151	0.046	2.613	1.130-6.468	0.029
Tumor location in bronchi						
Fourth-order bronchi	Ref.					
Fifth-order or higher bronchi	0.711	0.310-1.659	0.421			
CTR						
≤ 0.75	Ref.			Ref.		
>0.75	3.4	1.181-12.472	0.037	3.428	1.196-12.540	0.035
Pleural indentation						
Absence	Ref.			Ref.		
Presence	4.642	1.966-12.169	<0.001	4.441	1.899-11.499	0.001
CEA						
≤5.0 ng/ml	Ref.			Ref.		
>5.0 ng/ml	6.437	2.502-17.001	<0.001	6.521	2.537-17.196	<0.001

Ref., reference; OR, odds ratio; CI, confidence interval.

### 3.4 Model validation

**Figure 4:**
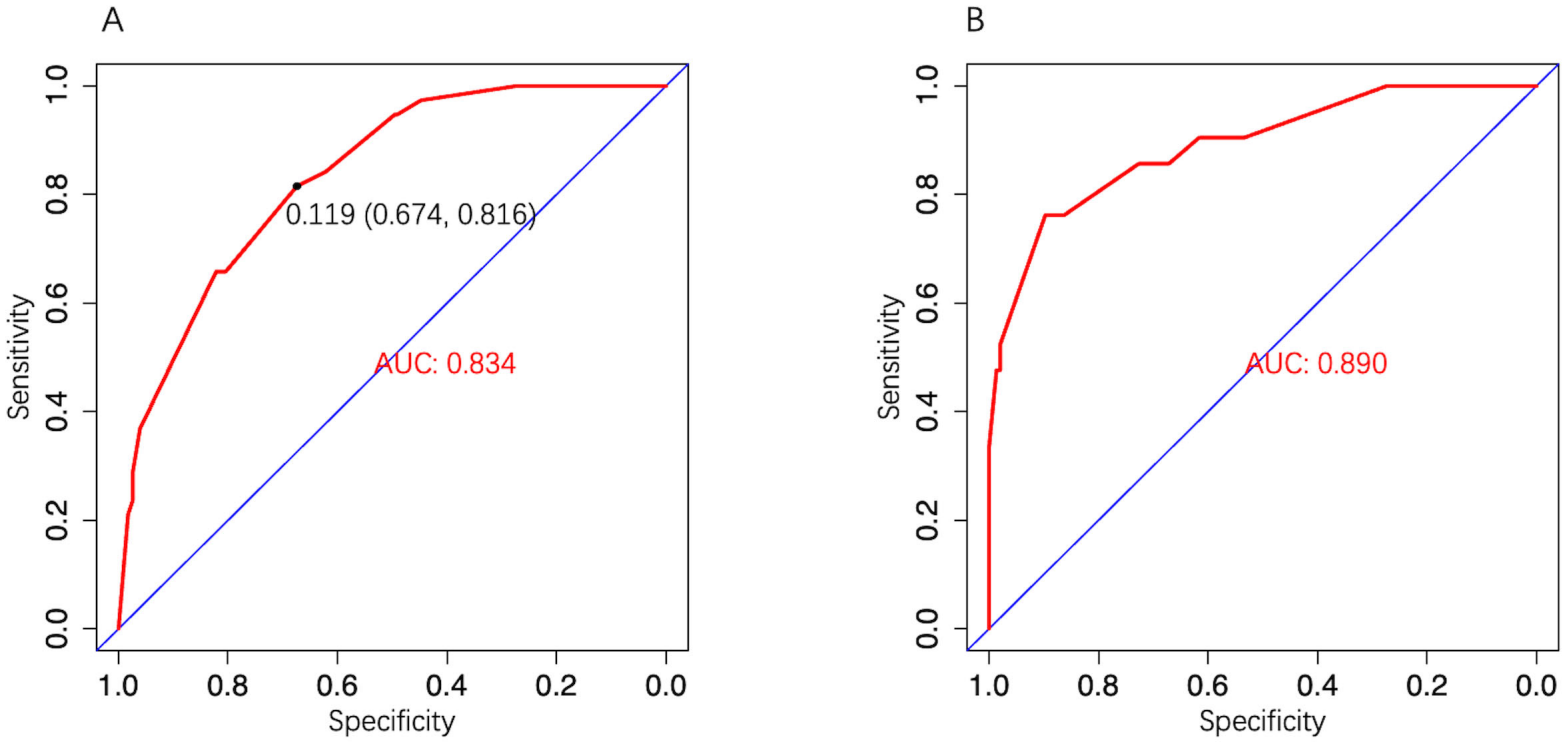
The model ROC curve. **(A)** Development cohort; **(B)** Validation cohort.

**Figure 5:**
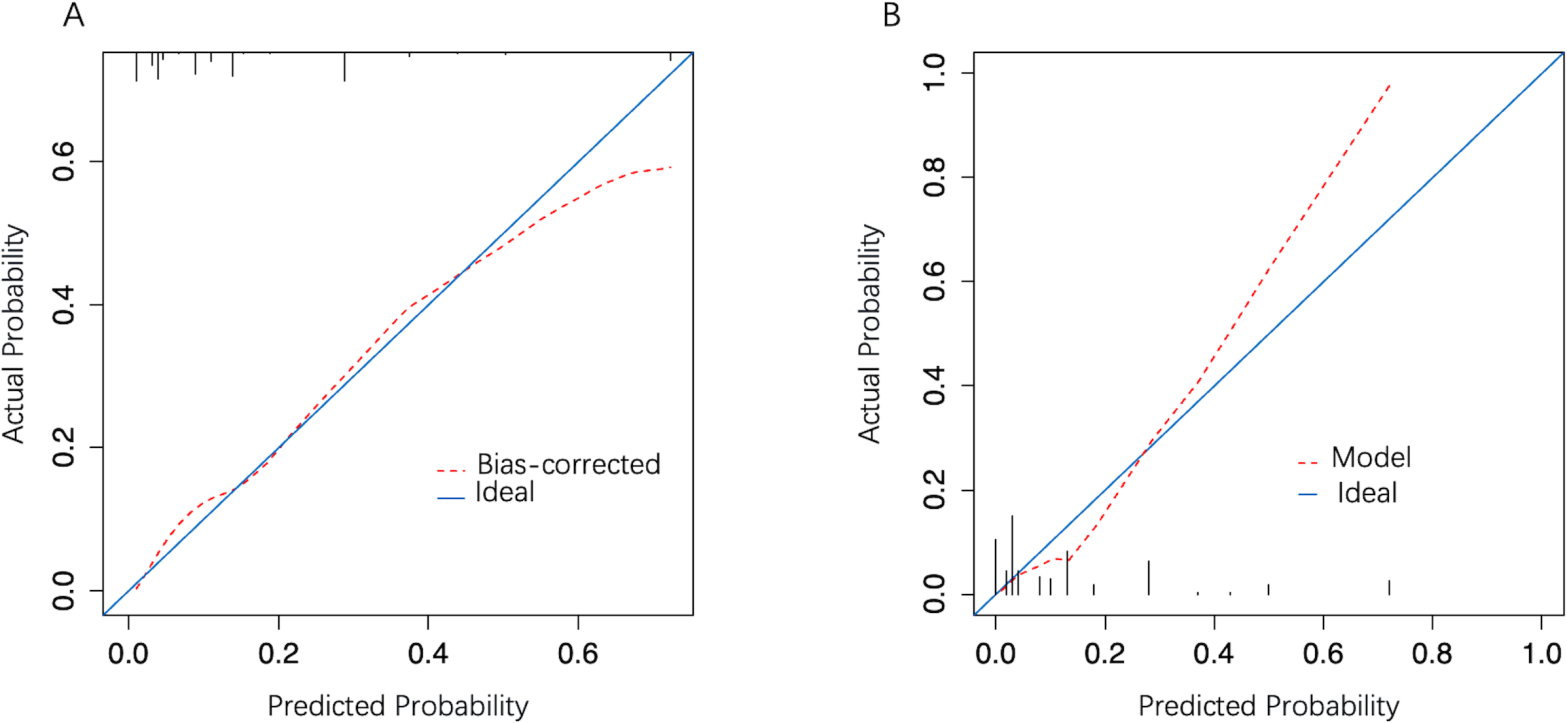
The model calibration curve. **(A)** Development cohort; **(B)** Validation cohort. The x-axis represents the predicted probability and the y-axis represents actual LNM probability. A perfect prediction corresponds to the diagonal solid blue line. The red dashed line represents model performance, of which, a closer fit to the diagonal line represents a better prediction.

In the development cohort, the model AUC was 0.834 (95% CI: 0.773–0.896) (**Figure 4A**), and when LNM-positive probability (11.9%) corresponding to the maximum Youden index (0.49) was used as a cutoff probability, model sensitivity was 81.6% (95% CI: 69.3%–93.9%) and specificity was 67.4% (95% CI: 61.3%–73.4%). HL tests showed that the model was well calibrated (P value = 0.921). The calibration curve also showed good agreement between predicted and actual results (**Figure 5A**). The model Brier score was 0.095.

**Figure 6:**
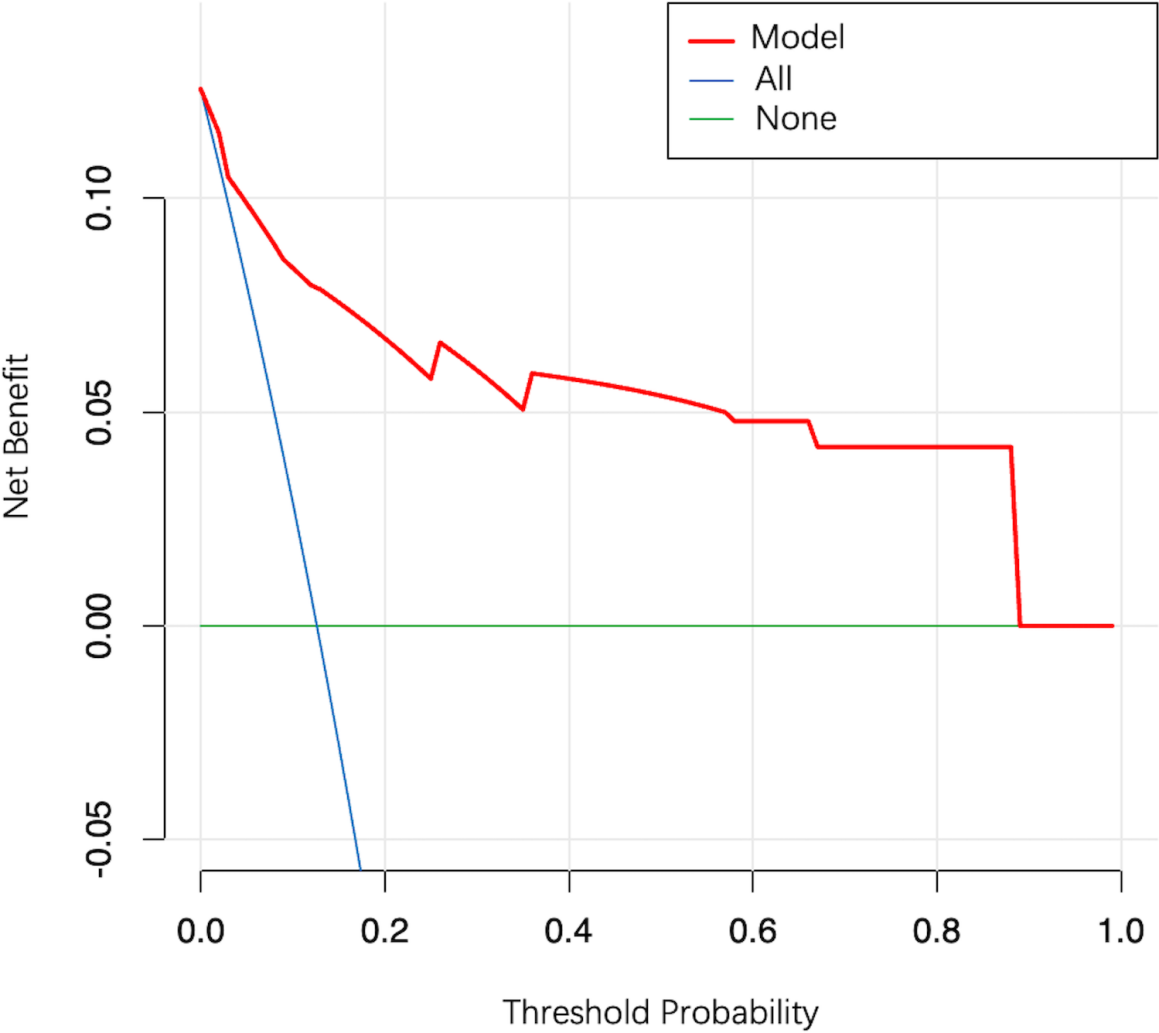
Decision curve analysis for the model. The y-axis measures the net clinical benefit. The red line represents the model. The blue line represents the assumption that all patients have LNM. The green line represents the assumption that all patients have no LNM.

In the external validation cohort, model AUC was 0.890 (95% CI: 0.808–0.972), demonstrating robust discriminatory ability (**Figure 4B**). When 11.9% was used as the model cutoff probability, corresponding sensitivity was 85.7% (95% CI: 71.4%–100.0%) and specificity was 72.6% (95% CI: 65.1%–80.1%). HL tests demonstrated good calibration (P value = 0.680). Although the calibration curve showed that agreement between predicted and actual results decreased when compared with the development cohort, it was still acceptable (**Figure 5B**), further illustrating the reliability of the calibration. The model Brier score in the external validation cohort was 0.065, which indicated an overall good performance. DCA showed that when using the model to predict LNM-positive probability, it delivered significantly more net benefits than other diagnostic options (**Figure 6**).

### 3.5 Risk stratification strategies based on clinical guideline recommendations and model performance

Based on high-risk characteristics as recommended by guidelines, we identified nine high-risk patients (5.4%) in the validation cohort. Of these, five were LMN-positive, resulting in a sensitivity of 23.8% and a specificity of 97.3% for the guideline strategy (**Table 5**). The diagnostic performance of different model strategies was as follows (**Table 5**); Strategy 1: Minimum model sensitivity was 33.3% (95% CI: 14.3%–57.1%), corresponding to a cutoff probability of 61.2% and a specificity of 100.0% (95% CI: 100.0%–100.0%); Strategy 2: The cutoff probability corresponding to the maximum Youden index was 11.9%, and corresponding model sensitivity was 85.7% (95% CI: 71.4%–100.0%) with a specificity of 72.6% (95% CI: 65.1%–80.1%); Strategy 3: When model sensitivity was elevated to > 90.5% (95% CI: 76.2%–100.0%), the corresponding specificity was 61.6% (95% CI: 53.2%–69.9%).

**Table 5:** The diagnostic performance of different strategies.

Diagnostic performance	Guideline strategy	Model strategy 1	Model strategy 2	Model strategy 3
Sensitivity	23.80%	33.3% (95%CI, 14.3%-57.1%)	85.7% (95%CI, 71.4%-100.0%)	90.5% (95%CI, 76.2%-100.0%)
Specificity	97.30%	100.0 (95%CI, 100.0%-100.0%)	72.6% (95%CI, 65.1%-80.1%)	61.6% (95%CI, 53.2%-69.9%)

CI, confidence interval.

## 4 Discussion

In this retrospective study, we developed and validated a predictive model to assess LNM-positive risk in peripheral NSCLC patients with a tumor size ≤ 2.0 cm and CTR > 0.5. The model indicated that patients with a tumor size > 1.5 cm, a CTR > 0.75, the presence of a pleural indentation, and a CEA value > 5.0 ng/ml had a greater risk of being LNM-positive.

Preoperative LNM status assessment is crucial for appropriate surgical choices in early-stage NSCLC patients. The much-anticipated JCOG0802 study (2022) required that patients with potential segmentectomy indications fulfilled applicable LNM-negative conditions (6). The CALGB140503 study (2023) (24), which was another heavyweight study, further side-stepped the importance of being LNM-negative for patients with indications for sublobar resection while obtaining similar results to the JCOG0802 study. Based on such clinical needs, we conducted this study with specific patient types similar to the JCOG0802 study and identified results that differed somewhat to the previous literature.

We found that tumor size was an independent predictor, consistent with most studies (25–29). Asamura and colleagues (26) reported that mediastinal LNM incidences in peripheral NSCLC patients increased from 19.5% for tumors ≤ 2.0 cm to 32.5% for tumors of 2.0–3.0 cm. Yang et al. (27) also reported that the probability of being LNM-negative was 70.8%, 58.88%, 48.03%, 47.55%, and 33.33% when tumor diameters were ≤ 2.0 cm, 2.1–3.0 cm, 3.1–5.0 cm, 5.1–7.0 cm, and > 7.0 cm, respectively. From further analyses, we found no interactions between tumor size and CTR (P value = 0.97).

Our results suggested that a higher CTR was an independent risk factor. A Japanese study reported that only 2% of adenocarcinoma patients with no solid component in their tumor had LNM, whereas 14% with a solid tumor component had LNM (30). Pathology-based studies also suggested that solid and sub-solid components roughly corresponded to invasive and non-invasive tumor components, respectively (31, 32). Since a higher CTR reflected a higher percentage of invasive components in adenocarcinoma (31, 32), and the majority of patients (95.6%) in our study had a pathological adenocarcinoma type, the association between higher CTR and higher LNM risk is reasonable.

Previous studies have been controversial with respect to pleural indentation as a radiographic indicator of tumor infiltration (20, 33, 34). Although some studies concluded that pleural indentation was associated with visceral layer invasion (20) and LNM (34), other studies concluded the opposite (33). It is possible that such contradictions were caused by subjective differences between researchers in determining pleural indentation. For this reason, we arranged for two specialists to carefully interpret CT images and examine interobserver agreements on result assessments to minimize errors. The findings conclusively suggested that the presence of a pleural indentation increased the risk of being LNM-positive, even for tumors ≤ 2.0 cm. Thus, these observations must be seriously considered by clinicians.

We observed no correlation between age and LNM. Previous studies reported that younger patients were more likely to have mutations in target genes (35), and that genotypic mutations were associated with LNM (36, 37), which may explain why a younger age is a risk factor for LNM in many studies (11, 25, 38). However, in the literature, any correlation between age and LNM was weakened or disappeared with decreasing tumor size when the study population was limited to NSCLC patients with tumors ≤ 3.0 cm (21, 33, 39), which we corroborated. Thus, we posited several plausible reasons for this: (I) a significant correlation between genotypic mutations and age no longer exists in small-sized tumors due to tumor heterogeneity and (II) interactions may occur between age and tumor size in predicting LNM risk. Further studies are required to clarify these reasons.

Many studies have demonstrated that central tumors have higher LNM rates than peripheral tumors (9, 23), which is consistent with the clinical logic that the closer the tumor is to the center, the more likely it is to invade lymph nodes in the drainage area. However, as no uniform standard exists for defining central versus peripheral lung cancer, study criteria often vary (2, 29, 40–42). Also, due to tumor heterogeneity (43), it is worth exploring whether the relationship between tumor location and LNM status is still applicable in small-sized peripheral tumors. Therefore, we attempted to define the tumor location based on the bronchial level at which the tumor is located and classified it with fourth-order bronchi (subsegmental bronchi) and fifth-order or higher bronchi (beyond the subsegmental bronchi). Such a definition could objectively reflect lesion proximity to the center, while at the same time refining classification and possibly detecting correlations at more subtle levels. We observed that patients with tumors at lower order bronchi were more likely to be LNM-positive (development cohort: 39.5% versus 30.9%, and validation cohort: 52.4% versus 34.9%), although this difference was not statistically significant (P value > 0.05). Thus, tumor location may not significantly affect LNM probability in these patients.

Our study had the following advantages over previous studies: (I) We validated our model using external data, thus model performance was more accurately evaluated; (II) We analyzed relationships between tumor location and LNM at more detailed levels based on the bronchial level and obtained the results; and (III) Our model is one of the few current models based on JCOG0802 study patients, and has better applicability when compared to other models.

Our study also has limitations. Firstly, an inherent retrospective bias may have occurred but we improved this using multivariate analysis. Secondly, we did not include maximum standardized uptake positron emission tomography-computed tomography (PET-CT) values as predictors because PET-CT is not a routine examination for small-sized NSCLC in clinical practice, and variables with more missing values are unsuitable for analysis. Finally, we were unable to enter N1/N2 LNM predictions separately due to our limited sample size. In future studies, we will improve this by collecting more samples.

In conclusion, we developed and externally validated a parsimonious clinical prediction model for calculating LNM-positive probability in patients with peripheral NSCLC, with a tumor diameter ≤ 2.0 cm and CTR > 0.5. Patients with a tumor size > 1.5 cm, a CTR > 0.75, a pleural indentation around the tumor on CT imaging, and a CEA value > 5.0 ng/ml were associated with an increased probability of being LNM-positive. The model showed good performance and has great potential in assisting clinicians making individualized clinical decisions.

## Data Availability

The raw data supporting the conclusions of this article will be made available by the authors, without undue reservation.
